# A Model System in S2 Cells to Test the Functional Activities of Drosophila Insulators

**Published:** 2015

**Authors:** M. Tikhonov, N. B. Gasanov, P. Georgiev, O. Maksimenko

**Affiliations:** Institute of Gene Biology Russian Academy of Sciences, Vavilova str., 34/5, 119334, Moscow, Russia

**Keywords:** insulator, copia enhancer, Su(Hw), enhancer transcription, hsp70 promoter

## Abstract

Insulators are a special class of regulatory elements that can regulate interactions between enhancers and promoters in the genome of high eukaryotes. To date, the mechanisms of insulator action remain unknown, which is primarily related to the lack of convenient model systems. We suggested studying a model system which is based on transient expression of a plasmid with an enhancer of the *copia *transposable element, in Drosophila embryonic cell lines. We demonstrated that during transient transfection of circle plasmids with a well-known Drosophila insulator from the *gypsy *retrotransposon, the insulator exhibits in an enhancer-blocking assay the same properties as in Drosophila stable transgenic lines. Therefore, the Drosophila cell line is suitable for studying the main activities of insulators, which provides additional opportunities for investigating the functional role of certain insulator proteins.

## INTRODUCTION

In cells of higher eukaryotes, an enhancer can activate a promoter at a distance of up to several hundred kilobase pairs [1-3]. The investigation of insulators may make a significant contribution to the understanding of the mechanisms of long-range interactions between regulatory elements. Insulators are regulatory elements capable of blocking the interaction between an enhancer and a promoter when located between them [4-7]. However, insulators do not directly affect the activity of the enhancer and promoter; i.e., the promoter can be activated by another enhancer, and the enhancer can activate another promoter. Recently, it became obvious that many insulator proteins provide specific interactions between distant regulatory elements and the structural domains of chromosomes [1].

Model systems derived from mammalian [8] and Drosophila [9-11] cell lines play an important role in the study of transcription factors acting as part of insulators. One of the problems in developing a convenient model system for the investigation of insulators is the relatively small number of described enhancers that are able to function effectively in Drosophila cell cultures.

**Fig. 1:**
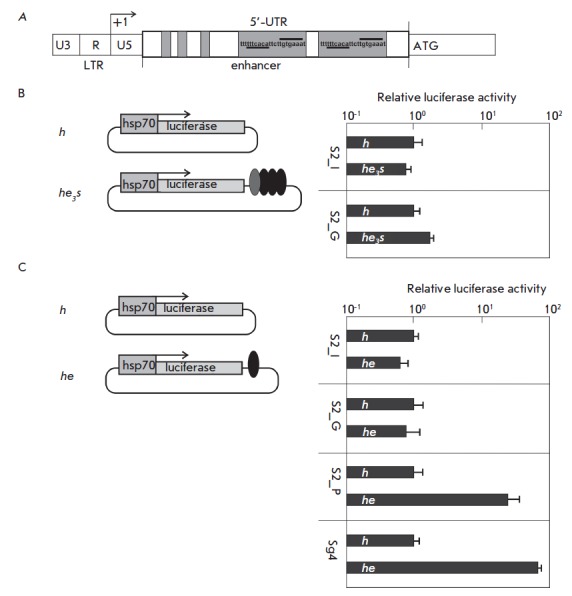
*A *– schematic diagram of an enhancer from the *copia *retrotransposon. The enhancer is located in the 5’-untranslated region (5’-UTR). LTR – long terminal repeat. +1 – transcription start. ATG – start codon. Gray rectangles denote octanucleotide repeats. *B *– results of an analysis of the activity of an element consisting of three copies of the *copia* enhancer (black ovals) and one copy of the SV40 enhancer (gray oval) located at the 3’-end of the firefly luciferase reporter gene (gray box), which is under control of the *hsp70*-promoter (gray rectangle with an arrow). The control construct *h *and tested construct *he3s *were transfected into two variants of S2 cells (S2_I and S2_G). The histogram presents, in a logarithmic scale, the firefly luciferase to jellyfish luciferase activity ratio. All data were normalized relative to the control construct *h*. The standard deviations were calculated on the basis of measurements of four biological replicates. *C *– analysis of the activity of one copy of the *copia *enhancer (black oval) located at the 3’-end of the firefly luciferase reporter gene. The control *h *and tested construct *he *were transfected into four variants of S2 cells (S2_I, S2_G, S2_P, and Sg4). The histogram presents, in a logarithmic scale, the firefly luciferase to jellyfish luciferase activity ratio. All data were normalized relative to the control construct *h*. The standard deviations were calculated on the basis of measurements of four biological replicates

An enhancer from the *copia *retrotransposon was previously shown to activate a promoter of the heat shock protein 70 gene in S2 cells from *Drosophila melanogaster*, having embryonic origin [10]. The 150 bp enhancer is located immediately after a 5’-long terminal repeat of the *copia *retrotransposon (*Fig. 1A*) and contains a 28 bp duplication at the 3’-end [12, 13]. The duplicated sequence comprises two copies of a TTGTGAAA octanucleotide in the inverted orientation. Three similar octanucleotides are located in the 5’-region of the enhancer. *Copia*-elements are known that contain an enhancer with only one 28 bp sequence and have a significantly reduced transcriptional activity. It is assumed that the TTGTGAAA sequence binds to a transcription factor, which determines the enhancer activity. Several transcription factors were also isolated that preferentially bind to the 5’-region of the enhancer and can both activate and inhibit transcription [13-15].

This work provides a detailed analysis of the *copia* enhancer in a model system that is used to test insulators in the Drosophila cell culture. The adequacy of the model system based on transient expression of circular plasmids in Drosophila cell cultures was studied using an insulator localized in the regulatory region of the Drosophila *gypsy *retrotransposon [4-7]. Previously, the basic properties of regulatory elements of this class were described with an example of this insulator using model systems based on Drosophila stable transgenic lines. The present work demonstrates that all basic properties of the *gypsy *insulator are reproduced during transient expression of a circular plasmid in a Drosophila cell culture.

## MATERIALS AND METHODS

**Development of constructs**

Plasmids pGL3basic and pGL3enhancer (Promega) were used as initial vectors. The *hsp70 *gene promoter (–203… + 253 bp relative to the transcription start) was amplified with *D. melanogaster *genomic DNA and inserted at the restriction sites HindIII and EcoRI into the pGL3basic and pGL3enhancer vectors. The 168 bp* copia *enhancer was amplified with *D. melanogaster* genomic DNA and inserted into the pGL3basic and pGL3enhancer vectors (*he *construct) downstream of the polyadenylation signal at the BamHI restriction site. Constructs *e_d_*and *e_r_*were prepared by inserting the amplified *copia *enhancer upstream of the luciferase gene coding region. In the constructs *e_d_h *and *e_r_h*, the *copia *enhancer was cloned into the *h *vector upstream of the promoter, at the SmaI restriction site. In the case of the constructs *g_d_e_d_h*, *g_r_e_d_h*, *g_d_e_r_h*, *g_r_e_r_h*,* e_d_g_d_h*, *e_d_g_d_h*, *g_d_e_d_g_d_h*, *g_r_e_d_g_d_h*, *e_d_s_d_h*, *e_d_s_d_g_d_h*, *e_d_s_d_g_r_h*, and* e_d_g_d_s_d_h*, a sequence of regulatory elements was first constructed on the basis of the pBluescript vector, and then the sequence was transferred to the *h *vector at the SmaI restriction site, upstream of the promoter. The *gypsy *insulator (from MDG4 retrotransposon) was a 450-bp fragment previously amplified in our laboratory. The SV40 virus polyadenylation signal was cut out from the pAc5.1hisB vector (Invitrogen) at restriction sites BamHI and SalI. In the case of constructs* he_d_g_d_*, *he_d_g_r_*, *hg_d_e_d_*, *hg_d_e_d_g_d_*, and *hg_d_e_d_g_r_*, a set of regulatory elements was also assembled in the pBluescript vector and transferred to the *h *vector at the BamHI restriction site, upstream of the polyadenylation signal. In the *g_d_hg_d_e_d_* construct, regulatory elements were inserted at the restriction sites SmaI and BamHI, upstream and downstream of the transcription unit, respectively.

**Cell culturing and transfection**

The *Drosophila *S2 cell culture was grown in a SFX medium (HyClone) at 25 °C. Cells were transfected with a Cellfectin II reagent (Invitrogen) according to the manufacturer’s recommendations (about 8 × 10^5^ cells per transfection). Two hours before transfection, the cells were put into wells of a 12-well plate. 0.5 μg of DNA was used for one transfection. In all cases, co-transfection of the tested constructs (the firefly luciferase gene was used as a reporter gene) and a control construct (the jellyfish luciferase gene was under control of the actin gene promoter at a 1 : 19 ratio) was performed. The cells were harvested 48 h after transfection.

**RNA extraction and reverse transcription**

RNA was isolated from S2 cells using a TRI-reagent (Ambion) according to the manufacturer’s recommendations. The isolated total RNA was purified from genomic DNA using a Turbo DNA-free reagent kit (Ambion). 1–5 μg of a RNA sample was mixed with a hexamer randomized primer (with a final concentration of 1–5 μM), heated to 70 °C, incubated for 5 min, and rapidly cooled in ice. Then, dNTPs at a concentration of 0.5 mM, buffer for reverse transcriptase, 5 units of the SUPERase-In RNase inhibitor (Ambion), and 60 units of ArrayScript Reverse Transcriptase (Ambion) were added. The reaction mixture was incubated at 42 °C for 2 h, then the enzymes were inactivated by heating to 95 °C for 5 min.

**Quantitative real-time PCR**

Quantitative real-time PCR was carried out in cDNA samples. Simultaneously, at least three independent reactions with each primer pair for each of three independently collected samples were conducted. Relative amounts of DNA were determined by ΔΔCt. Fragments of the *γTub37C *and *rpl32 *genes were used as an endogenous control. The following primer pairs were used in the study: tub (gctttcccaagaagctcataca and ggttcagtgcggtattatccag), rpl32 (gttcgatccgtaaccgatgt and ccagtcggatcgatatgctaa), Fluc (ttgctccaacaccccaacat and ttccgtgctccaaaacaaca), Rluc (cagtggtgggccagatgtaaacaa and taatacaccgcgctactggctcaa).

**Dual luciferase assay**

The dual luciferase assay was performed using a Firefly & the Renilla Luciferase Assay Kit (Biotium) according to the manufacturer’s protocol. The measurement was conducted on a microplate analyzer with a sensitivity of 100 and 1 s exposure time.

## RESULTS AND DISCUSSION

**Activity of an enhancer of the copia mobile element depends on the Drosophila cell line**

It was previously shown [10] that the *copia *enhancer (*Fig. 1A*) could cause a more than 100-fold increase in transcription from the promoter of the heat shock protein 70 (hsp70) gene of a plasmid transfected into S2 cells. However, according to [16], the *copia *enhancer does not stimulate transcription in S2 cells and its activity is detected only in the DH-33 cell line derived from *Drosophila hydei*.

The first possible explanation for these contradictory results was an assumption that the construct used in [10] contained additional regulatory elements that might have increased the *copia *enhancer activity in S2 cells. Indeed, an expression vector contained three copies of the *copia *enhancer at the 3’-side of the firefly luciferase reporter gene, which was controlled by the minimal *hsp70*-promoter (*Fig. 1B*). The SV40 (s) enhancer was located near *copia *enhancer copies (e_3_) and could also participate in the stimulation of transcription [10].

To study the role of the complex organization of the enhancer region in the stimulation of transcription, we compared the activity of this construct (*he3s*) and that of a construct containing only a promoter (*h*) in S2 cells from two different sources (*Fig. 1B*). One cell line was maintained in our laboratory (2S_G), and the second line was received from Invitrogen (2S_I). Surprisingly, a complex element consisting of three *copia *enhancers and the SV40 enhancer was found not to stimulate the* hsp70*-promoter in any of the tested cell lines (*Fig. 1B*). Thus, the complex enhancer does not stimulate transcription in S2 cells.

These results may be explained by the differences in the set of transcription factors that are expressed in S2 cell lines independently cultivated for a long time. To test this assumption, a new vector was generated that contained only one copy of the *copia *enhancer downstream the reporter gene (*Fig. 1C*). We used two additional cell lines: S2_P (line used in the MODEncode project) and Sg4 (received from Pirrotta’s laboratory, Rutgers University, USA). The Sg4 line is derived from the S2 line and differs from S2 in the expression profile of several genes.

On the basis of transfection of the control and tested plasmids into four cell lines, the *copia *enhancer was found to cause an approximately 80- to 100-fold increase in *hsp70*-promoter transcription in the Sg4 and S2_P lines, but not to have a stimulatory potential in two previously used S2 lines. Thus, one copy of the *copia* enhancer can efficiently stimulate transcription only in certain types of S2 cells.

**The copia enhancer induces bidirectional transcription with an efficiency comparable to the hsp70 promoter baseline activity**

In study [10], it was shown that a complex regulatory element consisting of SV40 and *copia *enhancers induces bidirectional transcription. Currently, there is abundant data showing that transcription initiation occurs on most enhancers [2, 3]. Short unstable non-polyadenylated transcripts are most often transcribed from enhancers. Usually, the transcripts are not transported into the cytoplasm and not translated. So, we decided to test the *copia *enhancer ability to induce transcription. Some enhancers were previously shown to be capable of producing full-length mRNAs [2, 3]. Therefore, the* copia *enhancer ability to produce polyadenylated and translated RNA was studied.

**Fig. 2:**
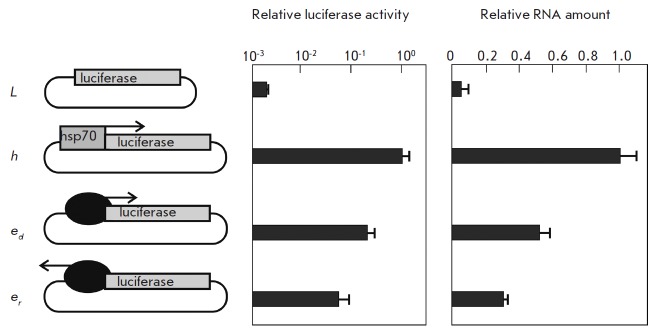
Results of the analysis of the promoter activity of the *copia *enhancer (black oval). The arrow shows the enhancer orientation. Plasmids *L *(negative control, no promoter) and *h *(positive control with the *hsp70*-promoter) were used as a control. The left histogram presents, in a logarithmic scale, the firefly luciferase to jellyfish luciferase activity ratio. All data were normalized relative to the control construct *h*. The standard deviations were calculated on the basis of measurements of four biological replicates. The right histogram presents, in a linear scale, the relative amount of RNA transcribed from the firefly luciferase gene. All data were normalized relative to the expression levels of the *rpl32*, *tub*, and luciferase jellyfish genes. The standard deviations were calculated on the basis of measurements of four biological replicates

For this purpose, a construct was generated where the *copia *enhancer was introduced in the direct or reverse orientation instead of the *hsp70*-promoter, upstream of the firefly luciferase reporter gene (*Fig. 2*). Plasmids with/without the *hsp70*-promoter upstream of the reporter gene were used as a control. These plasmids were used to transfect Sg4 cells. It was shown that the *copia *enhancer was able to start bidirectional transcription and expression of the luciferase, but 5–20 times less intensively compared to the construct with the *hsp70*-promoter. In the direct orientation, the *copia* enhancer acts as a promoter which is about 3 times stronger than in the reverse orientation. Thus, the *copia* enhancer can act as a weak bidirectional promoter inducing the formation of functional mRNA, which is used as a template for luciferase synthesis. The level of transcripts synthesized from the *copia *enhancer and *hsp70*-promoter were compared by reverse RNA transcription, followed by quantitative PCR. Transcription from the promoter was found to be only 2–3 times more efficient than transcription from the enhancer. Thus, one copy of the *copia *enhancer can trigger bidirectional synthesis of RNA molecules suitable for passing through translation stages, and at a level comparable to the baseline activity of the *hsp70*-promoter.

**An insulator from the gypsy retrotransposon has little effect on the activity of the copia enhancer located prior to a promoter**

The strongest Drosophila insulator consisting of 12 binding sites of the Su(Hw) protein is located in the regulatory region of the *gypsy *retrotransposon [17-19]. The activity of the insulator in Drosophila transgenic lines depends on tested enhancers and promoters. For example, one copy of the insulator completely blocks the activity of *yellow *gene enhancers, but it has almost no influence on the *white *gene enhancer activity [20, 21]. By means of transfection of a circular plasmid into S2 Drosophila cells, it was shown [9] that one copy of the *gypsy *insulator placed before a reporter gene promoter causes a two-fold reduction in the activity of the *copia *enhancer introduced into the 3’-side of the gene. The two-fold reduction may be explained by the insulator influence on both the enhancer activity and the promoter located nearby. For example, the Su(Hw) protein is detected not only on an insulator, but also on the sequences of the *copia *enhancer and *hsp70*-promoter in the transfected constructs [22].

**Fig. 3:**
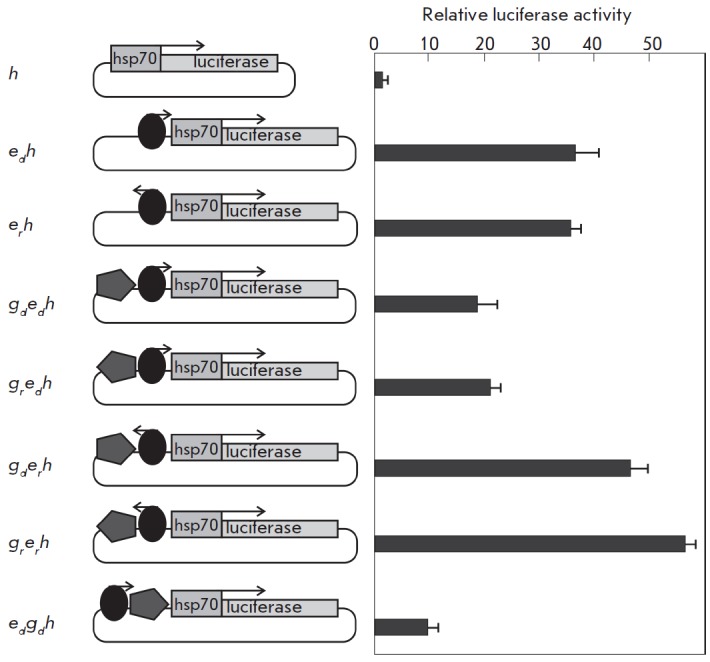
Effect of the *gypsy* insulator on the *copia *enhancer activity. The results of the analysis of the activity of combinations of the enhancer (oval) and the insulator (pentagon) located upstream of the *hsp70 *promoter are shown. The *copia *enhancer orientation is indicated by an arrow, and the insulator orientation is indicated by pentagon pointing. The histogram presents the firefly luciferase to jellyfish luciferase activity ratio. All data were normalized relative to the control construct *h*. The standard deviations were calculated on the basis of measurements of four biological replicates

To determine the element whose activity is affected by the insulator, we used a series of constructs with the enhancer at position –233 bp relative to the transcription start from the *hsp70*-promoter (*Fig. 3*). The enhancer was placed in two orientations: direct (*e_d_*) and reverse (*er*). The reporter gene expression level in transfected Sg4 cells was not dependent on the enhancer orientation. The insulator *gypsy *(*g*) was located immediately before the enhancer, in either direct or reverse orientation. Thus, four constructs were prepared in which the enhancer and insulator were placed in different orientations relative to each other and to the promoter. All the constructs were used to transfect Sg4 cells (*Fig. 3*). Determining the luciferase expression level demonstrated that the insulator orientation in constructs where the enhancer and the promoter had opposite orientations relative to each other did not affect, or slightly increased, the reporter gene expression level. In cases where the enhancer had a direct orientation, the reporter gene expression level was reduced approximately 2 times in the presence of the insulator in either orientation. Therefore, the insulator can affect the activity of the neighbor enhancer, which is located in close proximity to the promoter. In this case, the mechanism of influence is not associated with inhibition of interaction between the enhancer and the promoter. It is most likely that this orientation-dependent transcription inhibition is due to the direct interaction of proteins associated with the insulator and enhancer, which is consistent with the data on the distribution of insulator proteins [10].

The next task was to study the influence of the insulator on the expression level of the reporter gene at a position between the enhancer and promoter. For this purpose, we prepared a construct with the insulator inserted in position –233 bp relative to the transcription start of the *hsp70*-promoter (*Fig. 3*). The enhancer was located immediately before the insulator, in the direct orientation; i.e., the insulator was located between the enhancer and the promoter. In this case, the insulator reduced the enhancer activity by about 4 times. Thus, the insulator interposed between the enhancer and the promoter causes stronger inhibition of reporter gene transcription compared to the case where the insulator is upstream of the enhancer. This result is consistent with the basic property of insulators – the ability to block an enhancer – which is implemented when an insulator is interposed between an enhancer and a promoter.

**Two insulator copies surrounding an enhancer completely inactivate the enhancer activity**

**Fig. 4:**
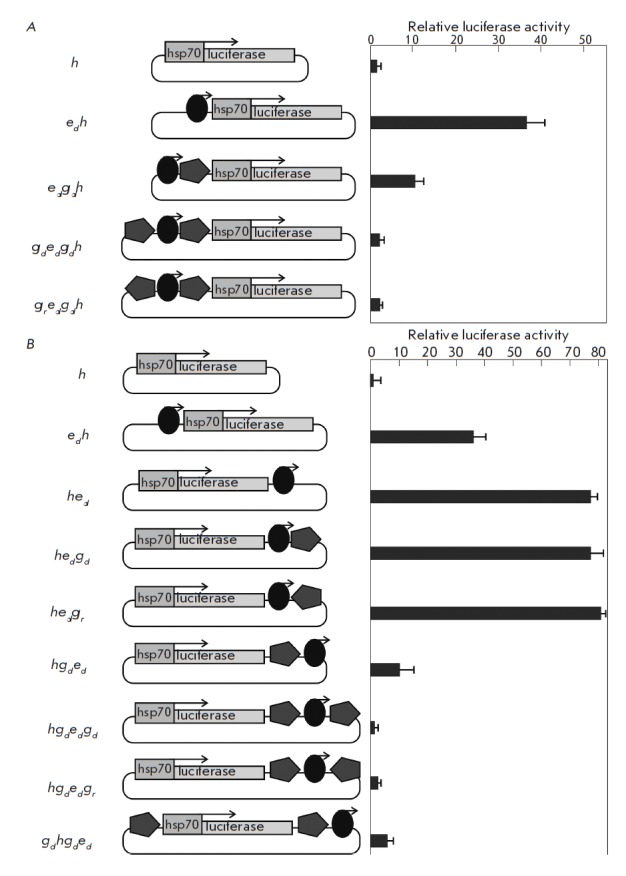
Effect of two *gypsy *insulator copies surrounding an enhancer or a reporter gene. Results of the analysis of combinations of two insulator copies (pentagon) in different orientations, which surround the enhancer (oval) and are located upstream of the *hsp*70 promoter (*A*) or at the 3’-end of the reporter gene, are shown (*B*). The *copia *enhancer orientation is indicated by an arrow, the insulator orientation is indicated by pentagon pointing. The histogram presents the firefly luciferase to jellyfish luciferase activity ratio. All data were normalized relative to the control construct *h*. The standard deviations were calculated on the basis of measurements of four biological replicates

The obtained results demonstrate that one copy of an insulator is only capable of partially blocking the enhancer activity in a transient model based on circular plasmids. Previously, we demonstrated that only two* gypsy *insulator copies surrounding either the enhancer or the *white *reporter gene are able to completely block the enhancer activity in Drosophila transgenic lines [21]. According to the model, the interaction between insulators leads to the formation of a chromatin loop, which greatly complicates interactions among the protein complexes associated with enhancers and promoters. To determine whether this rule of functioning of insulators works in the transient model based on a circular plasmid, two additional constructs were generated in which the enhancer located before the *hsp70*-promoter was surrounded by two insulators arranged in one or opposite directions (*Fig. 4A*). The reporter gene expression level in both variants was found to be close to the level of a control plasmid which contained only the *hsp70*-promoter. Thus, two insulators surrounding an enhancer lead to complete inactivation of its activity, which is consistent with the results obtained previously in transgenic Drosophila lines.

In the above-mentioned experiments, insulators surrounding the enhancer were located near the promoter. A question arises as to whether the effect of full enhancer inhibition is retained if an enhancer surrounded by insulators located at a considerable distance from the promoter. To answer this question, a number of constructs were generated in which the *copia *enhancer was inserted in the direct orientation at position +2,230 bp relative to the transcription start of the firefly luciferase reporter gene (*Fig. 4B*). At this position, the enhancer stimulated reporter gene transcription about twice more efficiently than at the position before the promoter. The reporter gene expression level in two plasmids in which the insulator was in the direct/reverse orientation relative to the 3’-side of the enhancer was close to the expression of a plasmid containing an enhancer only. Thus, the insulator located after the enhancer did not affect its activity. However, when the insulator was located between the reporter gene and the enhancer, a six-fold reduction in the reporter gene expression level occurred. Thus, the mutual arrangement of the insulator and the enhancer relative to the promoter even in a circular plasmid determines the efficiency of transcription inhibition. In the next series of constructs, the enhancer was inserted between two unidirectional or bidirectional insulators (*Fig. 4B*). Transient transfection of these plasmids into Sg4 cells caused reporter gene expression at the level of a control plasmid containing the *hsp70*-promoter only. Thus, two insulator copies surrounding the enhancer completely block its activity. Therefore, the distance between the enhancer and the promoter does not affect efficiency in blocking the enhancer interposed between a pair of insulators.

In transgenic Drosophila lines, two insulator copies surrounding the reporter gene caused weaker inhibition of the enhancer activity than two insulator copies surrounding the enhancer [21]. To further test the degree of correlation of the results obtained in circular plasmids and transgenic Drosophila lines, we used a construct in which insulators surrounded the reporter gene, and the enhancer was located immediately after the insulator, on the 3’-side of the gene. Sg4 cells transfected with this plasmid were detected with a weak enhancer activity, which is consistent with the assumption that insulators in this configuration are unable to completely block the enhancer. Complete enhancer inactivation was observed only when two insulators were located immediately next to the enhancer. Therefore, a complete correlation between the results obtained in transgenic Drosophila lines and in a transient model in Sg4 cells was found.

**Transcription from an enhancer regulates the gypsy insulator activity**

Previously, it was assumed [10, 23, 24] that transcription helps an enhancer move along chromatin in search of a promoter. According to this model, an insulator blocks promotion of the enhancer together with RNA polymerase II towards the promoter. Transcription, which is initiated on the enhancer, may also directly affect the activity of the promoter and the insulator.

**Fig. 5:**
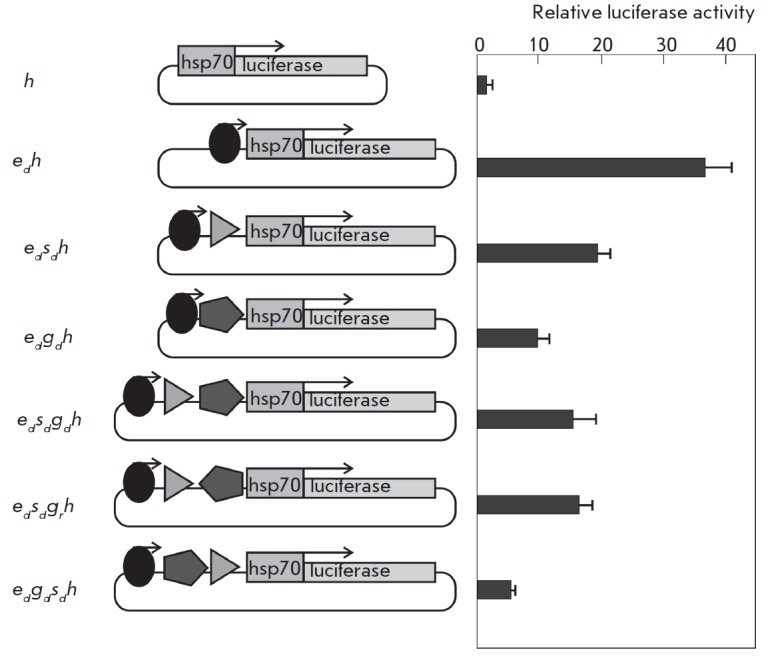
Effect of transcription from an enhancer on the *gypsy *insulator activity. Results of the analysis of combinations of the insulator (pentagon) in different orientations, enhancer (oval), and SV40 virus transcription terminator (triangle) located upstream of the *hsp70 *promoter are shown. The *copia *enhancer orientation is indicated by an arrow; the insulator orientation is indicated by pentagon pointing; the SV40 terminator orientation is indicated by triangle pointing. The histogram presents the firefly luciferase to jellyfish luciferase activity ratio. All data were normalized relative to the control construct *h*. The standard deviations were calculated on the basis of measurements of four biological replicates

To investigate the functional role of transcription initiated on an enhancer, we generated a number of plasmids with the 220 bp SV40 virus universal polyadenylation signal used to terminate transcription. In the first plasmid, the SV40 terminator was inserted between the *copia *enhancer, which was positioned in direct orientation, and the *hsp70*-promoter (*Fig. 5*). Transfection of Sg4 cells with the plasmid caused a 2-fold reduction in the reporter gene expression level compared to a plasmid containing the enhancer only. This result may be partly explained by the fact that transcription initiated from the enhancer contributes to the reporter gene expression. The SV40 terminator stops this transcription and, thus, reduces the reporter gene expression level. However, we demonstrated above (*Fig. 2*) that luciferase expression from the *copia* enhancer is about 5 times lower than that from the* hsp70*-promoter. Therefore, the main possible explanation is related to the fact that the SV40 terminator is able to partially block the interaction between the enhancer and the promoter by stopping the movement of RNA polymerase II from the enhancer to the promoter. This interpretation is consistent with a model in which RNA polymerase II plays a certain role in signal transmission from the enhancer to the promoter [10, 24, 25].

In the other two plasmids, the enhancer was inserted in direct orientation relative to the promoter and was separated from the promoter by the SV40 terminator and the insulator, which was inserted in direct or reverse orientation (*Fig. 5*). When Sg4 cells were transfected with any of these plasmids, the reporter gene expression remained at the same level as that of a plasmid containing the SV40 terminator only. Interestingly, transcription in the presence of a combination of the insulator and the terminator reached a higher level compared to a plasmid containing the insulator only. Thus, the SV40 terminator partially suppresses the inhibitory activity of the insulator instead of the expected additive negative effect of the insulator and SV40 terminator on the reporter gene expression. When Sg4 cells were transfected with a plasmid with a reversed order of the insulator and the terminator, whereby the insulator occurred between the enhancer and the terminator, a reduction in the reporter gene expression level was observed (*Fig. 5*). These data suggest that transcription from the enhancer increases the insulator activity, which leads to more effective inhibition of the enhancer.

## CONCLUSION

The data obtained in this work suggest that embryonic Drosophila cell lines with a common origin differ in their expression levels of the transcription factors necessary for the functioning of the *copia *enhancer. Apparently, expression of other genes encoding transcription factors not essential for maintaining the cell line can vary in embryonic cell lines. Thus, cell lines, even with a common origin, can greatly vary in their sets of transcription factors and, as a consequence, in the functional activity of regulatory elements.

We developed a model system that makes it possible to study the activity of insulators in Drosophila embryonic cell lines. In the circular plasmid-based transient model, the most well-known insulator *gypsy *retains its basic properties described using model systems based on transgenic Drosophila lines [25]. One copy of the insulator blocks only partially the enhancer activity, whereas two copies surrounding either an enhancer or a reporter gene cause complete inactivation of the enhancer.

Recently, our laboratory demonstrated that transcription via an enhancer inhibits its activity [26]. In the present study, we found that the *copia *enhancer has the properties of a weak bidirectional promoter, and transcription from the enhancer can increase the enhancer-blocking activity of the MDG4 insulator. Indeed, there is data according to which binding of transcripts to the Su(Hw) complex can regulate insulator activity [27, 28].

## Abbreviations

S2: Drosophila embryonic cell line
Sg4: S2-derived cell line
bp: base pairs
hsp70 promoter: hsp70 gene promoter
SV40: simian virus 40 polyadenylation signal
